# Hand-Litters for the Army

**Published:** 1862-12

**Authors:** 


*Hand-Litters for the Army.*—Medical-Purveyor, Dr. Lamb, advertises proposals for furnishing the Government with five hundred hand-litters, (ambulance pattern,) with the privilege of increasing the number to one thousand, if the needs of the service require it. Five hundred are to be delivered within ten days after the award of the contract.

---

It is announced that Surgeon-General Hammond is engaged in preparing a work on Military Hygiene.

